# Evaluation of a *Salmonella* Strain Lacking the Secondary Messenger C-di-GMP and RpoS as a Live Oral Vaccine

**DOI:** 10.1371/journal.pone.0161216

**Published:** 2016-08-18

**Authors:** Cristina Latasa, Maite Echeverz, Begoña García, Carmen Gil, Enrique García-Ona, Saioa Burgui, Noelia Casares, Sandra Hervás-Stubbs, Juan José Lasarte, Iñigo Lasa, Cristina Solano

**Affiliations:** 1 Laboratory of Microbial Biofilms, Instituto de Agrobiotecnología (Idab), Universidad Pública de Navarra-CSIC-Gobierno de Navarra, Pamplona, Spain; 2 Recombina S. L. Mutilva, Navarra, Spain; 3 Program of Immunology and Immunotherapy, Center for Applied Medical Research (CIMA), Instituto de Investigación Sanitaria de Navarra (IdISNA), University of Navarra, Pamplona, Spain; 4 Navarrabiomed-Universidad Pública de Navarra, Instituto de Investigación Sanitaria de Navarra (IdISNA), Pamplona, Spain; New York Medical College, UNITED STATES

## Abstract

Salmonellosis is one of the most important bacterial zoonotic diseases transmitted through the consumption of contaminated food, with chicken and pig related products being key reservoirs of infection. Although numerous studies on animal vaccination have been performed in order to reduce *Salmonella* prevalence, there is still a need for an ideal vaccine. Here, with the aim of constructing a novel live attenuated *Salmonella* vaccine candidate, we firstly analyzed the impact of the absence of cyclic-di-GMP (c-di-GMP) in *Salmonella* virulence. C-di-GMP is an intracellular second messenger that controls a wide range of bacterial processes, including biofilm formation and synthesis of virulence factors, and also modulates the host innate immune response. Our results showed that a *Salmonella* multiple mutant in the twelve genes encoding diguanylate cyclase proteins that, as a consequence, cannot synthesize c-di-GMP, presents a moderate attenuation in a systemic murine infection model. An additional mutation of the *rpoS* gene resulted in a synergic attenuating effect that led to a highly attenuated strain, referred to as ΔXIII, immunogenic enough to protect mice against a lethal oral challenge of a *S*. Typhimurium virulent strain. ΔXIII immunogenicity relied on activation of both antibody and cell mediated immune responses characterized by the production of opsonizing antibodies and the induction of significant levels of IFN-γ, TNF-α, IL-2, IL-17 and IL-10. ΔXIII was unable to form a biofilm and did not survive under desiccation conditions, indicating that it could be easily eliminated from the environment. Moreover, ΔXIII shows DIVA features that allow differentiation of infected and vaccinated animals. Altogether, these results show ΔXIII as a safe and effective live DIVA vaccine.

## Introduction

*Salmonella* remains a foodborne pathogen of rising concern to consumers and governments worldwide. In Europe, *Salmonella* is the second most frequently reported cause of foodborne outbreaks with known origin, with *Salmonella enterica* sv *enteritidis* (S. Enteridis) and *Salmonella enterica* sv *typhimurium* (S. Typhimurium) being the two most commonly detected serovars. The number of officially reported clinical cases of salmonellosis amounts to almost 90.000 according to the report of the European Food Safety Authority (EFSA) for the year 2014 (https://www.efsa.europa.eu/en/efsajournal/pub/4329), and the overall economic burden has been estimated to be as high as 3 billion euros a year.

The fact that *Salmonella* gastroenteritis cases usually follow the consumption of contaminated basic food products such as poultry derivatives or pig meat, in combination with the rapid spread of multidrug resistant *Salmonella* spp. triggered by the high-productivity-focused model of animal breeding [1,2] has prompted the appearance of new policies aimed at the prevention of *Salmonella* intake into the food chain. Thus, public health programs including means such as the improvement of hygienic conditions in farms, the use of fodder supplements and the execution of effective vaccination protocols are being gradually implemented. Many *Salmonella* vaccines have been tested in poultry and swine, with varying degrees of success (for review, see [3–5]). These can be divided into three categories: live-attenuated, inactivated and subunit vaccines. As regards whole cell killed and subcellular vaccines, biosafety for both human and farm animals is their main advantage. However, it is generally accepted that protection conferred by these last preparations is fairly modest when they are compared to vaccines based on live attenuated *Salmonella* strains [6,7]. This assertion is supported by the potential of live bacteria to activate both humoral and cellular adaptive immune responses [8] and by their capacity to inhibit intestinal colonization during the “immunity gap” (period of time after neonatal vaccination when there is no longer sufficient maternal immunoglobulins to afford protection from infection but when there is still enough of this maternal protection to prevent the young animal from mounting its own protective immune response) [9]. If other evidences like the easiness of production and administration are considered, we obtain a scenario in which the livestock industry calls for new live attenuated vaccines that display an improved balance between attenuation (safety) and immunogenicity (efficacy).

Since the early 90´s, attenuation of *Salmonella* has been accomplished by the introduction of mutations in *aro* genes and global regulators like PhoPQ, Crp or RpoS (for review, see [10]). Other common attenuation strategies are based on auxotrophies raised by mutation of genes involved in the synthesis of purines (*purA*, *purE* mutants) or in the metabolism of carbohydrates (*galE* mutants) [11,12] and on the elimination of determinants directly involved in infection establishment (e.g. pathogenicity islands, virulence plasmid, *svp* genes) [13,14]. Present-day advances widening the knowledge about molecular mechanisms underlying *Salmonella* virulence and the development of novel DNA engineering tools are currently leading to more ambitious genetic approaches in the area of recombinant vaccines, and long-term visions include *Salmonella* strains with regulated delayed attenuation *in vivo* and their use as antigen carrier /delivery platforms [15]. Nevertheless, fairly curious is the fact that some of the strains used as commercial vaccines, such as SG 9R, VacT or VacE, have been generated by passages in the laboratory or by random mutagenesis and thus, the exact genetic basis of their attenuation is largely unknown [10].

In our laboratory, we constructed a *S*. Enteritidis strain, called ΔXII, carrying mutations in the twelve genes encoding GGDEF domain proteins and thus, incapable of synthesizing the secondary messenger bis-(3–5)-cyclic dimeric GMP (c-di-GMP) [16]. This molecule is widely recognized as a key regulator of bacterial biology, including the transition from a planktonic to a biofilm lifestyle and from the virulent state in acute infections to a less virulent state characteristic of chronicity [17]. Furthermore, bacterial cyclic nucleotides have been recently involved in modulating the innate immune response, leading to induction of type I interferons, via direct binding to the eukaryotic Stimulator of Interferon Genes (STING) and to DDX41 [18–21]. Virulence assays with *S*. Enteritidis ΔXII showed that it was highly attenuated in orally and intraperitoneally infected BALB/c mice [16] and hence, this strain might be considered a potential vaccine candidate. However, further work with ΔXII revealed that it contained an additional chromosomal deletion of 16.8 kilobases that included the *rpoS* gene [22]. Since RpoS, which is the master sigma factor during stationary phase and under a variety of stress conditions, plays a critical role in *Salmonella* virulence [23], it was unclear whether the absence of c-di-GMP played any role in virulence attenuation shown by ΔXII. To solve this and other questions related to c-di-GMP signaling, a new ΔXII strain was generated, sequenced and confirmed to only lack GGDEF proteins encoding genes [22].

Taking new ΔXII strain as a basis, the present work was planned with three main objectives: (i) analyzing the contribution to *Salmonella* virulence of c-di-GMP signaling; (ii) confirming the attenuation of a strain deficient in c-di-GMP signaling and RpoS with the aim of identifying a genetic background that might lead to a novel attenuated *Salmonella* vaccine strain and, (ii) assessing its potential use as an effective vaccine to combat gut and organ colonization by *Salmonellae*. Here, we report that total depletion of c-di-GMP in new ΔXII correlated with a moderate loss of virulence in a murine model of infection. A ΔXII strain in which we also performed a *rpoS* mutation led to a strain that we called ΔXIII, more attenuated than a single *rpoS* mutant but still capable of eliciting a cellular/humoral balanced, long-lasting immune response against a lethal oral-challenge of a *S*. Typhimurium virulent strain. Moreover, use of ΔXIII enabled serological differentiation of vaccinated and infected mice due to the lack of antibodies raised against the *Salmonella* specific GGDEF protein SEN4316 in vaccinated animals.

## Materials and Methods

### Ethics statement

Animal studies were performed in accordance with the European Community guiding in the care and use of animals (Directive 2010/63/EU). Protocols were approved by the ethics committee of the Public University of Navarra (Comité de Ética, Experimentación Animal y Bioseguridad of the Universidad Pública de Navarra) (approved protocol PI-004/11). Work was carried out in the animal facility of the Instituto de Agrobiotecnología, Universidad Pública de Navarra. Animals were housed under controlled environmental conditions with food and water ad libitum. Mice were euthanized by CO_2_ inhalation followed by cervical dislocation and all efforts were made to minimize suffering.

### Bacterial strains and culture conditions

Bacteria were grown in LB broth and on LB agar with appropriate antibiotics at the following concentrations: kanamycin (Km), 50 μg ml^-1^; ampicillin (Am), 100 μg ml^-1^; chloramphenicol (Cm), 20 μg ml^-1^ and apramycin (Apr), 60 μg ml^-1^.

Bacterial strains used in this study are listed in S1 Table. *S*. Enteritidis 3934 (MIC-54) is a wild type clinical isolate described in [24,25]. *S*. Enteritidis ΔXII (MIC-1324) is a multiple mutant, derivative of 3934, carrying mutations in all genes encoding GGDEF domain proteins and thus incapable of synthesizing c-di-GMP [22]. This multiple mutant was generated by a markerless gene replacement method described in [16,22] and as a consequence, does not contain any exogenous DNA. To compare the virulence of the wild type and ΔXII strain we used a ΔXII derivative (MIC-3664), resistant to kanamycin and tetracycline, that was generated in [22].

*S*. Typhimurium CNM 143/09 (MIC-1201) is a clinical strain isolated from a patient that suffered a pig meat borne gastroenteritis (Centro Nacional de Microbiología; Instituto de Salud Carlos III).

To generate the single mutant Δ*rpoS* (MIC-2101), a PCR-generated linear DNA fragment in combination with the helper plasmid pKOBEGA were used [25,26]. A selectable antibiotic resistance gene was generated by PCR using oligonucleotides rpoS-Apra_fw (ctgaaagttcatgatttaaatgaagacgcggaatttgatgagaacggagtagaggctttttcccatccaccggatcaatt) and rpoS-Apra_rv (tgcgctcgttgagacgaagcatacggctgacgtcatcaaccggtttatccagttgctctgtccttgttagacattatttg) that included 60-nt homology extensions for the targeted locus and 20-nt priming sequences for the apramycin resistance gene as template from a freshly isolated colony of *E*. *coli* MC4100 F´tet Δ*traD*::*aac* [27]. Electroporation (25 μF, 200 Ω, 2.5 kV) was carried out according to the manufacturer’s instructions (Bio-Rad) using 50 μl of cells and 1 to 5 μg of purified and dialyzed (0.025 μm nitrocellulose filters; Millipore) PCR product. Shocked cells were added to 1 ml of LB broth, incubated overnight at 30°C, and then spread on LB agar with Apr to select Apr^R^ transformants after incubation at 30°C for 24 h. Mutants were then grown on LB broth with Apr at 43°C for 24 h and incubated overnight on LB agar with Am at 30°C to test for the loss of the helper plasmid.

To generate the vaccine candidate, *S*. Enteritidis ΔXIII (MIC-1330), the *rpoS* gene was deleted in ΔXII (MIC-1324) strain as follows. DNA fragments corresponding to the upstream (fragment AB) and downstream (fragment CD) regions of the *rpoS* gene were amplified with the following oligonucleotides, with chromosomal DNA from strain *S*. Enteritidis 3934 as a template. Primers RpoS_1´_Fw (gaatcgtatacaatcgccag) and RpoS_B_XhoI (ctcgaggctcctacccgtgatcc) were used to amplify the AB fragment. Primers RpoS_C_XhoI (ctcgagttgtcaaaaaaaggccagtc) and RpoS_D_BglII (agatctaatctgccacaggtgatg) were used to amplify the CD fragment. The PCR products were cloned in the pGEMt-easy vector (Promega), digested with NotI and XhoI enzymes in the case of the AB fragment and XhoI and BglII enzymes in the case of the CD fragment, and ligated in the same ligation mixture with the pKO3blue vector [16] digested with NotI and BglII enzymes. The recombinant pKO3blue::AD vector was extracted from *E*. *coli* XL1 Blue and electroporated into strain *S*. Enteritidis ΔXII. The following steps of integration and excision of the plasmid were performed as described previously [16]. As a result, ΔXIII is a multiple mutant in all genes encoding GGDEF domain proteins and in *rpoS* and does not contain any exogenous DNA.

### Mice survival assays

Mice were acclimated for 7 days after arrival before the experiments were started. In the case of oral infections, food and water were removed, twelve and two hours respectively, before the administration of bacterial suspension. Mice were prefed with 20μl of 10% sodium bicarbonate 30 min before bacterial inoculation. Water and food were again supplied right after inoculation.

For mice survival assays, eight-week-old female BALB/c mice (Charles River Laboratories) were infected orally with 10^7^ *Salmonella* wild type or mutant cells that had been grown overnight in LB broth and resuspended in 100 μl of PBS. Mice survival was monitored for 20–28 days. Over the course of infection, mice were examined twice per day and a final disease score was given to each mouse according to clinical signs observed as follows. No clinical signs (0); mild clinical signs: ruffled fur (1); moderate clinical signs: ruffled fur plus, lethargy, hunched posture and decreased activity (2); severe clinical signs: paresis, paralysis, tremor, shivers, ataxia, rigidity (3). When evident signs of disease (score 2 to 3) were observed, mice were euthanized by CO_2_ inhalation followed by cervical dislocation.

### Mice competitive infections

Infection studies were carried out with 8-week-old female BALB/c mice (Charles River Laboratories). Mice were inoculated intraperitoneally (i.p.) or intragastrically (i.g.) with 100 μl of bacteria suspended in phosphate-buffered saline (PBS; pH 7.4). The total bacteria inoculum was 2x10^4^ cfu (i.p.) or 2x10^8^ cfu (i.g.) of combined *S*. Enteritidis wild-type and mutant strains at a ratio of 1:1. The cfu of each strain in the inoculum (input) was quantified by plating dilution series on LB agar and using antibiotic resistance to distinguish between strains. Mice were euthanized after 3 days (i.p.) or 5 days (i.g.), and dilution series of liver and spleen lysates were plated on LB agar for enumeration of cfu (output), using antibiotic resistance to differentiate strains. Values for competitive index (CI) were calculated as the ratio of wild type to mutant in the output divided by that in the input, and the CI was expressed as log_10_ [28].

To compare the *in vivo* interaction of *Salmonella* strains with murine intestinal epithelial cells, the ligated ileal loop co-infection model was used as described previously [25]. Values for CI were calculated as described above.

### Mice protection assay

Groups of seven 8-week-old female BALB/c mice were orally inoculated with 10^7^ cfu of the attenuated strains ΔXIII or ΔrpoS. One control group only received PBS. On day thirty-three after immunization, immunized and control animals were intragastrically challenged with 10^6^ cfu of virulent *S*. Typhimurium 143/09. Vaccinated and control mice were monitored daily to assess clinical signs as described above. When evident signs of disease (score 2 to 3) were observed, mice were opportunely euthanized by CO_2_ inhalation followed by cervical dislocation.

### Preparation of ΔXIII heat-killed lysate

The vaccine strain, ΔXIII, was cultured overnight at 37°C on LB agar plates. Bacteria were resuspended in PBS and the OD_600_ was adjusted to 0.2 (1x10^8^ cfu ml^–1^, based on viable cell counts on LB agar). Bacteria were then incubated at 75°C in a water bath for 30 min. The effectiveness of the inactivation was assessed by plating 100 μl of the suspension on LB agar, after treatment.

### Determination of antibody titers

Blood samples from the orbital vein of each mouse from the control and the ΔXIII-immunized groups were collected before immunization and 14 and 28 days after immunization. Samples were centrifuged and sera from each group were collected. Serum IgG and IgM were detected by ELISA. For that, Nunc Maxisorp 96-well plates (Thermo Fisher Scientific, San Jose, CA) were coated with 10^7^ cfu of heat-killed ΔXIII bacteria and incubated at 4°C overnight. Plates were then washed three times with PBS containing 0.05% Tween 20 (PBS-T; pH 7.4) and blocked with 5% nonfat dried milk powder in PBS-T at room temperature for 1 h. After three washes with PBS-T, immune mouse sera were serially diluted from an initial dilution of 1:20. A 100 μl volume of diluted sample was added to duplicate wells and incubated at 4°C overnight. Wells were washed three times with PBS-T and 100 μl of horseradish peroxidase (HRP)-conjugated goat anti-mouse IgG, IgM (H+L) secondary antibody diluted 1:2500 (Thermo Scientific) was added to each well. The plates were incubated for 1 h at room temperature and then washed three times. One hundred microliters of 3,3′,5,5′-Tetramethylbenzidine (TMB) (Sigma-Aldrich) were added to each well and the absorbance at 405 nm was determined on an ELISA reader. Endpoint titers were expressed as the reciprocal log_2_ values of the last positive sample dilution. Absorbance readings higher than the mean plus two standard deviations of preimmune serum baseline values were considered indicative of positive reactions.

### Opsonophagocytosis uptake assay

The murine macrophage-like cell line J774.2 was grown in RPMI 1640 with 10% heat-inactivated fetal bovine serum (FBS) and 1% Penicillin/Streptomicin (Lonza) at 37°C with 5% CO_2_. Cells were seeded in 24-well tissue culture plates. Once in confluence (0.2 x 10^6^ cells per well), cells were washed with PBS, and medium without FBS was added 16 hours prior to the experiment. Fourteen hours later, the medium was replaced by medium without antibiotics. 2 x 10^6^ cfu of an overnight culture of *S*. Typhimurium143/09 was mixed with 10% of pooled immune serum, pre-immune serum or PBS in 500 μl of Minimum Essential Medium Eagle (MEM) with 1% Bovine Serum Albumin (BSA) and complement (complement sera rabbit, Sigma-Aldrich). After incubation at room temperature for 15 minutes, the mix was added to the monolayer cells (multiplicity of infection of 10) and plates were incubated for 30 minutes at 37°C in 5% CO_2_. To kill extracellular bacteria, cells were washed and incubated for 90 minutes with 500 μl of RPMI 1640 containing 100 μg/ml gentamicin (Sigma-Aldrich). Cells were washed three times with sterile PBS and lysed with 0.1% Triton X-100. The number of phagocytosed bacteria was determined by plating serial dilutions of the lysates on LB agar and data were represented as relative bacterial uptake of bacteria mixed with serum with respect to the uptake of bacteria suspended in PBS, which was given a value of 1.0. Experiments were conducted in triplicate.

### ELISPOT assay

The number of IFN-γ producing cells was measured by ELISPOT using a kit from BD-Biosciences (BD Biosciences, San Diego, CA) according to the manufacturer´s instructions. Splenocytes were harvested from individual mice 28 days after immunization. Ninety-six-well Multi-Screen high protein binding Immobilon-P membrane plates (Millipore, Billerica, MA) were coated with mouse anti–IFN-γ capture antibody, incubated overnight, and blocked for 2 h with RPMI containing 10% fetal calf serum. Then, 1 x 10^6^ splenic cells in 10% fetal calf serum-RPMI were added to each well in triplicate and cultured in the presence or in the absence of 10^7^ cfu of heat-killed ΔXIII bacteria. Following overnight incubation, the plates were washed three times with PBS and incubated with biotinylated anti-IFN-γ antibody for two hours. Wells were washed three times and incubated with a solution of streptavidin-peroxidase for one hour. Then, plates were washed and developed with freshly prepared 3,3′-Diaminobenzidine (DAB) solution. The reaction was stopped with distilled water and spots were counted using an ELISPOT automated reader (CTL; Aalen, Germany).

### Measurement of cytokine production by ELISA

1 x 10^6^ splenic cells harvested from individual mice 28 days after immunization were co-cultured with 10^7^ cfu of heat-killed ΔXIII bacteria during 48 h. RPMI media was used as negative control. IL-2, IL-10 and IL-17 released to the supernatant were measured by ELISA using specific kits from BD Biosciences (San Diego, CA) according to the manufacturer´s instructions.

Levels of IFN-γ and IL-5 in serum were determined using a Mouse Procarta Cytokine 2Plex Assay (eBioscience). Levels of IL-10 in serum were determined with a Mouse IL-10 ELISA Ready-SET-Go!® kit (eBioscience).

### Flow cytometry analysis

Splenocytes (2x10^6^/well) harvested from individual mice 28 days after immunization were either stimulated with 10^6^ and 10^7^ cfu of heat-killed ΔXIII bacteria or rested unstimulated for 1h. Golgi-Plug and Golgi-Stop (BD Biosciences, San Diego, CA) were added and plates were incubated for five hours. Then, cells were washed with staining buffer (PBS, 0.5% BSA, 2 mM EDTA), surface stained with anti-CD8a-APC (53-6-7) and anti-CD4-FITC (RM45) monoclonal antibodies (mAbs), fixed and permeabilized with Cytofix/Cytoperm solution (BD Biosciences) and finally stained for intracellular cytokines with anti-TNF-α-PE-Cy7 (MP6-XT22) and anti-IFN-γ-PE (XMG1,2) mAbs. All mAbs were from Biolegend (San Diego, CA). Dead cells were excluded by FSC and SSC. Data were collected in a FACSCalibur cytometer (BD Biosciences) and analyzed using FlowJo software (Tree Star, San Carlos, CA).

### Biofilm formation and desiccation experiments

Biofilm formation in a rich-medium condition (LB) was determined and visualized as previously described [25]. Colony morphology and color on Congo red agar plates were tested after 48 hours of incubation at 28°C [29].

The desiccation experiment was adapted from a described protocol [30]. Briefly, 100 μl from overnight cultures grown in LB medium at 37°C were tested immediately (initial numbers) or air dried and stored in 24 well tissue culture plates at room temperature for 12 days. After rehydration of bacteria in 500 μl PBS, pH 7.4, the number of viable cells remaining in each sample was determined by serially diluting cell mixtures and plating in duplicate. The experiment was conducted in triplicate.

### Production of recombinant SEN4316

The *sen4316* gene was amplified from wild type genomic DNA with primers sen4316 BamHI-fw (ggatccatgacaacaccatcctggcg) and sen4316 SalI-rv (gtcgactcatagggcgcgcatgtcgt), using Phusion High-Fidelity DNA Polymerase (Fermentas-Thermo Scientific). The PCR-amplified fragment was cloned in pGEM-T Easy (Promega), sequenced and digested with BamHI and SalI to clone it into the pET28a vector (Novagen). The resulting plasmid pET28a::*sen4316* was electroporated into *E*. *coli* BL21 C43 (DE3) [31]. Cultures were grown at 37°C, 250 rpm, to an optical density (OD_600_) of 0.5, and isopropyl-D-thiogalactopyranoside (IPTG) was added to a final concentration of 0.4 mM. Cells were then grown overnight at 23°C. Harvested cells were lysed with BugBuster HT Protein Extraction Reagent (Merck Millipore). SEN4316 accumulated in inclusion bodies was obtained by centrifugation and suspension of insoluble material in CTAB 1%, incubation at room temperature for 1 h and recovery of the supernatant by centrifugation at 20,000 x g. This supernatant was dialyzed against binding buffer (20 mM sodium phosphate, 500 mM NaCl, 20 mM imidazole, pH 7.4) and the recombinant protein was purified with a His GraviTrap affinity column according to standard protocols (GE Healthcare). Eluted protein was dialyzed against sterile water, analyzed by SDS–PAGE and Western-Blot and lyophilized.

### SEN4316 based ELISA

Groups of ten mice were orally inoculated with a single dose of 10^7^ cfu or 10^4^ cfu of ΔXIII or the wild type strain, respectively. Blood samples from the orbital vein of each mouse were collected before inoculation and 33 and 44 days after inoculation. Samples were centrifuged and sera from each group were collected and pooled. Nunc Maxisorp 96-well plates (Thermo Fisher Scientific, San Jose, CA) were coated with SEN4316 (2 μg/well) in carbonate-bicarbonate buffer (pH 9.6) and incubated at 4°C overnight. Plates were then washed three times with PBS-T and blocked with 5% nonfat dried milk powder in PBS-T at room temperature for 2 h. After three washes with PBS-T, 100 μl of pooled sera diluted 1:100 in PBS-T containing 2.5% of BSA were added to each well and incubated at room temperature for 2 h. Wells were washed three times with PBS-T and 100 μl of horseradish peroxidase (HRP)-conjugated goat anti-mouse IgG, IgM (H+L) secondary antibody diluted 1:1250 (Thermo Scientific) was added to each well. The plates were incubated for 2 h at room temperature and then washed three times. One hundred microliters of ABTS (Sigma-Aldrich) were added to each well and the absorbance at 420 nm was determined on an ELISA reader.

### *sen4316* PCR to differentiate vaccinated from infected mice

Faecal samples from the wild type and ΔXIII infected groups were pooled and collected at day 1, 7, 14 and 21 post-infection. Cages were cleaned daily to remove non collected faeces. DNA from stool was extracted using a QIAamp DNA Stool Mini Kit (Qiagen). Oligonucleotides DIVA-1 (cacgattacgccaactcgagttgt) and DIVA-2 (gtaagataactgtgcgaag) were used to amplify a 632 bp fragment from ΔXIII DNA. Amplification of *invA* with oligonucleotides invA-fw (ggcgatattggtgtttatgg) and invA-rv (catattatcgctatcgccat) was used to amplify a 658 bp fragment from wild type and ΔXIII DNA. The PCR amplification conditions were as follows: 1 cycle at 95°C for 5 min; 30 cycles of the following: 94°C for 30 s, 54°C for 30 s and 72°C for 35 s; 1 cycle of 72°C for 7 min.

### Statistical analyses

All statistical analyses were performed in GraphPad Prism 5.01. A Log-rank (Mantel-Cox) test was used to assess significance in mice survival assays. A Wilcoxon signed-rank test was used to determine whether the CI values were significantly different from the hypothetical value of zero. A two-way analysis of variance combined with the Bonferroni test was used to analyze statistical significance in antibody titers, cytokine serum levels and data from flow cytometry assays. A nonparametric Mann-Whitney test was used to assess significant differences in the opsonization assay. Differences in cytokine production in splenocytes assayed by ELISPOT and ELISA were determined using the unpaired Student’s t test.

## Results

### Analysis of the contribution of c-di-GMP signaling to *Salmonella* virulence

**Fig 1:**
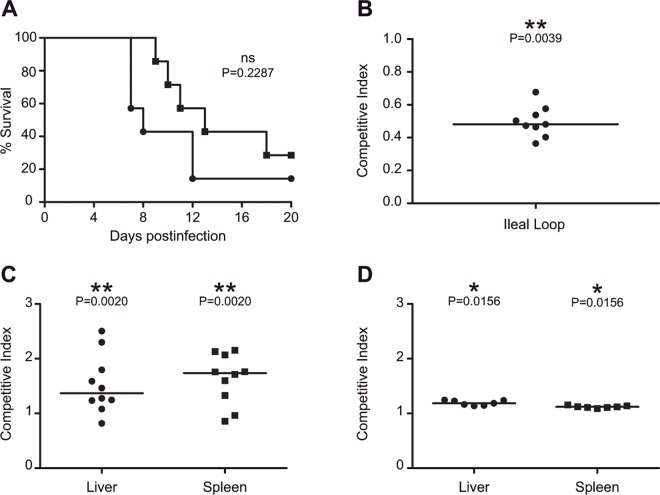
c-di-GMP signaling in *Salmonella* is required for intestinal epithelium and organ colonization of BALBc mice but its absence does not significantly affect BALBc mice survival. (A) Comparative lethality between wild-type (black circles) and ΔXII (black squares) strains in an oral infection mouse model. Inoculum administered was 10^7^ cfu/mouse. P-value was determined by a Log-rank (Mantel-Cox) test. ns; no significant difference. (B) Competitive index (CI) analysis of wild type and ΔXII strains after performing an ileal loop coinfection experiment. Nine ileal loops were coinfected with 2x10^7^ cfu containing equal numbers of the parental and ΔXII strains. (C) CI analysis following intragastric inoculation of ten BALBc mice with a 1:1 mixture of wild type and ΔXII strains (total inoculum administered was 2 x 10^8^ cfu). Mice were sacrificed after five days and bacteria were enumerated from livers and spleens. (D) CI analysis following intraperitoneal inoculation of seven BALBc mice with a 1:1 mixture of wild type and ΔXII strains (total inoculum administered was 2 x 10^4^ cfu). Mice were sacrificed three days postinoculation and bacteria were enumerated from livers and spleens. CI was defined as the log_10_ of the ratio of wild type strain to ΔXII strain recovered (Output) divided by the ratio of wild type strain to ΔXII strain present in the inoculum (Input). A CI > 0 indicates wild type with a colonization advantage compared to ΔXII and a CI < 0 indicates wild type with a colonization disadvantage over ΔXII. The plots display values obtained from individual samples and the median CI is represented by horizontal bars. P-values were determined by a Wilcoxon signed-rank test. *P < 0.05; ** P < 0.01; *** P < 0.001.

To evaluate the impact of the absence of c-di-GMP on *Salmonella* virulence, we firstly compared the survival rates of groups of seven BALB/c mice, which are susceptible to systemic infection with *Salmonella*, orally infected with 10^7^ cfu of the wild type *S*. Enteritidis strain 3934 or its derivative, new ΔXII strain. ΔXII showed virulence attenuation that manifested by a latter time to death than animals infected with wild type *Salmonella*. However, the difference between survival curves was no statistically significant (p = 0.2287) and at the end of the experiment ten and twenty percent of mice survived after infection with the wild type and ΔXII respectively (Fig 1A). Virulence of ΔXII after infection by the natural oral route was further investigated by carrying out a competitive index (CI) analysis in an ileal loop coinfection experiment and also, by assessing the level of organ colonization following oral co-inoculation of the wild type and ΔXII strains. ΔXII showed a significantly reduced capacity to adhere and invade the intestinal epithelium (Fig 1B) and a significantly lower ability to colonize livers and spleens of orally infected mice (Fig 1C). Defectiveness of ΔXII colonization at systemic sites was additionally confirmed by evaluating organ colonization after intraperitoneal (i.p) infection. ΔXII mutant colonized both the spleen and liver of i.p infected mice significantly less than the wild type strain (Fig 1D). Taking together, these results showed that c-di-GMP signaling is required for natural virulence of *Salmonella* in BALB/c mice, since absence of c-di-GMP rendered bacteria significantly less able to adhere and invade the intestinal epithelium and to colonize systemic organs during acute infection. However, the overall outcome of the lack of c-di-GMP signaling in pathogenicity is subtle, resulting in a non significant virulence attenuation.

### Construction and virulence characterization of a *Salmonella* strain lacking all GGDEF proteins and the RpoS sigma factor

**Fig 2:**
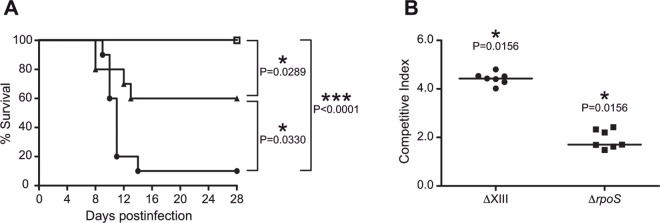
The synergic effect of the absence of RpoS and c-di-GMP signaling in ΔXIII strain results in high virulence attenuation. (A) Comparative lethality between wild-type (black circles), ΔrpoS (black triangles) and ΔXIII (open squares) strains in an oral infection mouse model. Groups of ten mice were orally infected with 10^7^ cfu/mouse. P-values were determined by a Log-rank (Mantel-Cox) test. (B) CI analysis following intragastric inoculation of seven BALBc mice with a 1:1 mixture of wild type and ΔXIII or ΔrpoS strains (total inoculum administered was 2 x 10^7^ cfu). Mice were sacrificed after seven days and bacteria were enumerated from spleens. CI for the wild type and mutant strains was defined as stated in Fig 1 legend.

Results described above carried out with new ΔXII strain, which only lacks GGDEF proteins encoding genes, demonstrated that absence of c-di-GMP has a restrained impact on virulence. On the other hand, the firstly described ΔXII strain, that also contains a chromosomal deletion that includes the *rpoS* gene, is completely avirulent in mice [16]. Hence, we next wondered whether this avirulent phenotype was solely caused by the absence of RpoS or by the synergic effect of the lack of RpoS and c-di-GMP. To analyze this, we compared the pathogenic behavior of the wild type strain, a single *rpoS* mutant, and a derivative of new ΔXII strain in which we deleted the *rpoS* gene. This novel mutant was named ΔXIII. A survival experiment of orally infected BALB/c mice with 10^7^ cfu of each strain showed that, in agreement with our previous results, ΔXIII strain exhibited a total attenuated behavior and thus, all mice infected with this strain survived at day 28 and did not present any morbid symptoms (Fig 2A). In contrast, the survival rate of mice infected with the wild type strain was only 10% at the end of the experiment, whereas infection with the *rpoS* mutant rendered a final survival of 60% of infected mice (Fig 2A). To certify virulence loss of ΔXIII, mice inoculated with this strain were sacrificed at the end of the experiment and a complete clearance of infection was confirmed by the absence of *Salmonella* in livers, spleens and faeces.

Since a *Salmonella* vaccine strain needs to be invasive enough to induce durable immunity in the host and thus, an excessive attenuation of ΔXIII strain was not desirable, we tested its invasiveness capacity at an earlier time of infection. For that, we orally infected groups of seven BALB/c mice with 2x10^7^ cfu of a 1:1 mixture of the wild type and Δ*rpoS* or ΔXIII strains. At day 7 post infection, animals were euthanized, bacterial load in spleens was determined and the CI was calculated. As shown in Fig 2B, ΔXIII was capable of reaching and colonizing internal organs, although, when compared to the wild type strain, it presented an approximately 10^4^ fold defect in its ability to colonize spleens of infected mice. Again, Δ*rpoS* mutant exhibited an intermediate attenuation phenotype, confirmed by a 10^2^ deficiency in spleen colonization.

Altogether, these data show that in ΔXIII strain, the absence of both c-di-GMP signaling and the sigma factor RpoS exerts a synergistic attenuating effect on *Salmonella* virulence, that turns this novel mutant strain into a potential live attenuated vaccine.

### Evaluation of the protection conferred by vaccination with ΔXIII

**Fig 3:**
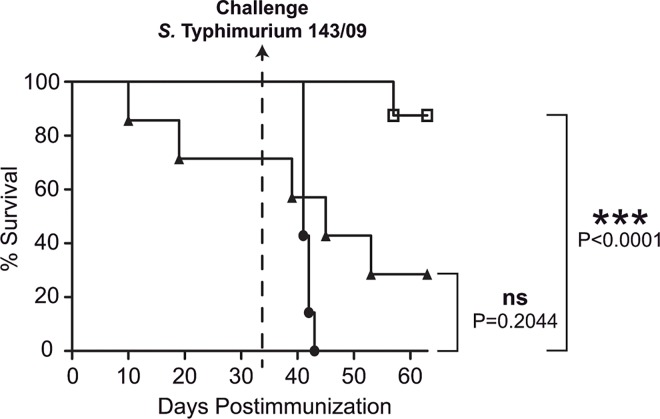
Vaccination-challenge analysis of the protection conferred by ΔXIII. Groups of seven BALBc mice were orally vaccinated with 10^7^ cfu of ΔXIII (open squares), Δ*rpoS* (black triangles) or PBS as a control (black circles). 33 days post immunization, mice were challenged with 10^6^ cfu/mouse of *S*. Typhimurium 143/09. Survival curves were plotted and P-values were determined by a Log-rank (Mantel-Cox) test.

With the aim of investigating the efficacy of ΔXIII strain as an orally administered vaccine against salmonellosis, a vaccination-challenge analysis was carried out in BALBc mice, using a clinical isolate of *S*. Typhimurium as the challenge strain. The reason why this strain was used for challenge is that, according to the report of the European Food Safety Authority (EFSA) for the year 2014 (https://www.efsa.europa.eu/en/efsajournal/pub/4329), *S*. Enteritidis and *S*. Typhimurium are the two most commonly reported serovars in confirmed human cases. Moreover, *S*. Enteritidis is the second most commonly reported serovar from broiler meat and *S*. Typhimurium is the most frequently reported serovar in pigs and cattle and also in pig meat. By using *S*. Typhimurium for challenge we assessed both ΔXIII vaccination efficacy and also the level of cross-protection against other *Salmonella* serovar. Groups of 7 mice were orally immunized with a single dose of 10^7^ cfu of either the ΔXIII or ΔrpoS strain. This last strain was used as an immunizing control. A third group of mice was given 100 μl of sterile PBS. Vaccinated and control untreated mice were challenged 33 days post immunization with 10^6^ cfu (10 fold the LD_50_) of *S*. Typhimurium 143/09 and data on number of dead mice were daily collected. As shown in Fig 3, all non vaccinated animals died within a 10 day period post challenge. In contrast, 90% of ΔXIII immunized mice survived after challenge. Vaccination with Δ*rpoS* resulted much less effective, firstly because, according to the results presented above, 30% of mice died before challenge, and secondly, because, after challenge, only 30% of initial mice remained alive. These results demonstrated that immunization with ΔXIII confers a very significant degree of crossprotection against salmonellosis by *S*. Typhimurium in mice.

### Characterization of the immune response generated by immunization with ΔXIII strain

**Fig 4:**
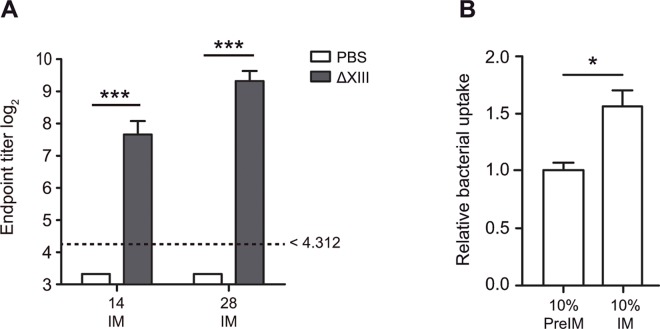
Humoral immune responses in mice immunized with ΔXIII strain. A) Sera obtained 14 and 28 days post-immunization from mice immunized with ΔXIII present significantly higher levels of IgG and IgM against heat-killed ΔXIII bacteria than sera from non immunized mice (control, treated with PBS) as determined by ELISA. No antibody to heat-killed ΔXIII bacteria was detected in sera from control mice (reciprocal titer, <1:20). Error bars represent standard deviation between individual antibody titers. Statistical analysis was carried out using a two-way analysis of variance combined with the Bonferroni test. *** P < 0.001. B) Opsonization with immune serum enhances the uptake of *Salmonella* by murine macrophage cells. Opsonization and phagocytosis of *S*. Typhimurium143/09 by J774.2 macrophages was tested by counting the number of phagocytosed bacteria that had been previously mixed with 10% immune serum obtained 28 days post immunization (IM), pre-immune serum (PreIM) or PBS. The relative bacterial uptake of bacteria mixed with serum with respect to the uptake of bacteria suspended in PBS (value of 1.0) is represented. Results from duplicates on three separate days are shown. Statistical analysis was performed using the Mann–Whitney test. *P < 0.05.

While performing the vaccination experiments described above, and in order to know the basis of the protection conferred by ΔXIII, serum from mice of the control and ΔXIII immunized groups were collected right before immunization and 14 and 28 days after immunization. An ELISA assay was then carried out to measure the levels of IgG and IgM against a ΔXIII heat-killed lysate in individual sera. Results showed a very significant antibody response in mice immunized with ΔXIII strain when compared to control mice, treated with PBS (Fig 4A). Then, pooled sera samples were used to examine the efficiency of such generated antibodies to promote opsonization and phagocytosis of *S*. Typhimurium 143/09 by J774.2 murine macrophage cells. As shown in Fig 4B, the number of phagocytized bacteria was significantly higher when bacteria were preincubated with 10% of sera from ΔXIII immunized animals than when incubated with preimmune sera. The specificity of these antibodies against *Salmonella* was confirmed by the fact that the uptake of bacteria preincubated with preimmune sera was very similar to the uptake of bacteria suspended in PBS (relative bacterial uptake close to one).

**Fig 5:**
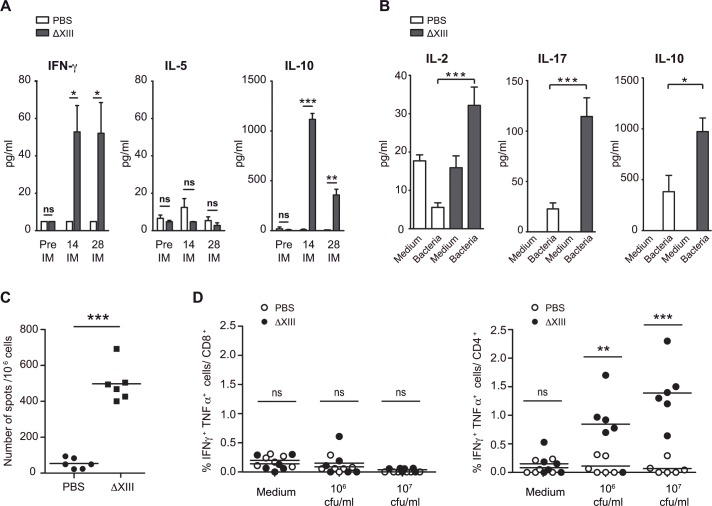
ΔXIII induced cellular immune responses. A) Sera from ΔXIII immunized mice present significantly higher levels of interferon gamma (IFN-γ) and IL-10 than sera from the control group, treated with PBS. Quantification (pg/ml) of IFN-γ, IL-5 and IL-10 in pooled sera from the control group (PBS) and from the ΔXIII immunized group was carried out using respective ELISA kits. Error bars represent standard deviation between duplicate wells. Statistical analysis was carried out using a two-way analysis of variance combined with the Bonferroni test. ns = no significant difference; *P < 0.05; ** P < 0.01; *** P < 0.001. B) ΔXIII immunization induces production of cytokines in splenocytes. 28 days postimmunization, splenocytes were harvested from control and ΔXIII immunized mice and restimulated for 48 h with 10^7^ cfu of heat-killed ΔXIII bacteria. RPMI media was used as a non-stimulating control. Cell supernatants were harvested and analyzed for IL-2, IL-10 and IL-17 production using respective ELISA kits. Statistical analysis was carried out using the unpaired Student *t* test. C) Numbers of IFN-γ producing cells in spleens from control and ΔXIII immunized mice were determined by ELISPOT assay. Splenocytes were harvested from all mice at 28 days after immunization. The results are expressed as spots per million splenocytes minus background from cells stimulated with 10^7^ cfu of heat-killed ΔXIII bacteria. Each point represents an individual mouse. Lines indicate the mean of the replicates. Significant differences between groups are indicated and were determined using the unpaired Student *t* test. *** P < 0.001. D) CD4+ T cells produce IFN-γ and TNF-α upon ex vivo re-stimulation following vaccination with ΔXIII. 28 days after immunization, splenocytes from control and ΔXIII immunized mice were plated and stimulated for 6 hours with medium, 10^6^ or 10^7^ cfu of heat-killed ΔXIII bacteria. Cells were surface stained with anti-CD4 and anti-CD8 mAbs and intracellularly stained with anti-IFN-γ and anti-TNF-α antibodies. Shown are percentages of IFN-γ and TNF-α cells out of total CD4+ and CD8+ T cells. Each point represents an individual mouse. Lines indicate the mean of the replicates. Significant differences between control and vaccinated groups are indicated and were determined using the unpaired Student *t* test. ns = no significant difference; ** P < 0.01; *** P < 0.001.

Since both humoral and cellular responses are necessary for immunity to oral challenge with virulent *S*. Typhimurium [7], we next determined serum levels of IFN-γ (a prototype Th1 cytokine), as well as IL-10 and IL-5 (prototype Th2 cytokines). Remarkably, significant IFN-γ and IL-10 levels were detected in pooled sera from ΔXIII immunized animals and not from PBS treated mice (Fig 5A). In particular, 14 days after immunization, IL-10 levels in sera from ΔXIII immunized mice were much higher than in sera from control mice (p<0.001). These levels decreased over time, although 28 days after immunization, they were still significantly higher than in control sera (p<0.01). As regards IFN-γ, it was also significantly present in sera from ΔXIII immunized mice (p<0.05) collected 14 and 28 days after immunization. On the other hand and in the case of IL-5, it is important to note that this cytokine was barely detectable in all samples analyzed.

To confirm the results obtained from sera and to evaluate the ΔXIII specific stimulation of cytokines production by spleen cells from immunized mice, we performed the following experiments. Supernatants of stimulated splenocytes with 10^7^ cfu of heat-killed ΔXIII bacteria were analyzed for the production of IL-2 (prototype Th1 cytokines), IL-10 (prototype Th2 cytokines), and the Th17-associated cytokine IL-17. Remarkably, levels of the three cytokines were significantly higher in supernatants from splenocytes of immunized mice than in supernatants from splenocytes of control mice (Fig 5B). It is important to note that differences were higher in the case of IL-2 and IL-17 production (p<0.001) than in the case of IL-10 (p<0.05). Also, an ELISPOT assay was carried out to investigate IFN-γ production and results showed that splenic lymphocytes from mice immunized with ΔXIII strain produced significantly more levels of IFN-γ secreting T cells than those of control mice (p<0.001) (Fig 5C). Finally, we used flow cytometry coupled with stimulations with heat-killed ΔXIII bacteria to measure IFN-γ and TNF-α production by CD4 and CD8 T cells (Fig 5D and S1 Fig). A strong CD4 T cell response specific of ΔXIII heat-killed bacteria was detected in AXIII immunized mice. Curiously, IFN-γ and TNF-α production was not detected in CD8 T cells (Fig 5D, left). It is likely that the use of heat-killed bacteria for the intracellular detection of cytokines might have hindered the accurate analysis of the CD8 T cell response, since heat-killed bacteria are not efficiently processed and presented by MHC-I molecules [32]. The assessment of the CD8 T cell response induced by ΔXIII requires further work that involves the identification of bacterial antigens and/or the use of recombinant bacteria expressing reporter antigens.

Overall, these data indicate that mechanisms underlying ΔXIII mediated protection include the production of opsonizing antibodies and a mixed T cell response characterized by significant levels of IFN-γ, TNF-α, IL-2, IL-17 and IL-10.

### Evaluation of ΔXIII as a live DIVA vaccine

**Fig 6:**
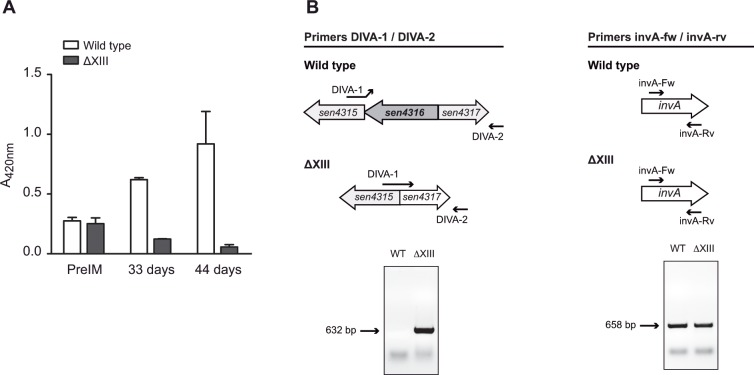
ΔXIII is a DIVA vaccine that allows differentiation of infected and vaccinated animals. (A) SEN4316 based ELISA of pooled sera from wild type and ΔXIII infected animals, obtained before infection (PreIM) and 33 and 44 days post infection. Sera from animals infected with the wild type strain and not with ΔXIII present antibodies against the SEN4316 protein. Error bars represent standard deviation between triplicate wells. (B) DNA from pooled faecal samples from wild type and ΔXIII infected animals, collected at day 1, 7, 14 and 21 post infection, were analyzed by PCR. Amplification of stool DNA with primers DIVA-1 and DIVA-2 allowed the identification of vaccinated animals with ΔXIII strain. Amplification with primers invA-fw and invA-rv served as a control of the presence of *Salmonella* DNA. Results shown are representative of results obtained throughout time, since *Salmonella* DNA was present in faecal samples since day one post infection.

A *Salmonella* ideal vaccine must not interfere with control programs, in the sense that infected animals with virulent field *Salmonella* strains must be differentiated from vaccinated animals. Therefore, we next aimed at exploring the properties of ΔXIII strain as a DIVA (Differentiation of Infected and Vaccinated Animals) vaccine. Since ΔXIII strain is a multiple mutant carrying deletions in *rpoS* and also in all GGDEF domain proteins encoding genes, we investigated the possibility of using the absence of one GGDEF protein as a DIVA marker. Interestingly, the GGDEF domain protein encoding gene *sen4316* does not show any orthologous gene in other enterobacteriaceae and thus, we found conceivable the idea of using the antibodies raised against this protein, that could only be developed in animals that had been colonized by a natural wild type strain but would be absent in vaccinated ones, in order to differentiate infected and vaccinated individuals. To prove so, groups of 10 mice were orally inoculated with a single dose of 10^7^ cfu of ΔXIII or a sublethal dose (10^4^ cfu) of the wild type strain and sera samples were obtained periodically up to day 44 post inoculation. Concurrently, we designed a specific ELISA assay in which a 6-His–tagged recombinant version of SEN4316 was used as the bound antigen. This assay showed that titers of antibodies against SEN4316 in pooled sera from animals infected with the wild type strain rose gradually after infection. On the contrary, and as expected, titers in pooled sera proceeding from ΔXIII immunized animals remained negative (Fig 6A).

In addition to the immunoassay, we also explored the possibility of using the *sen4316* gene as a positive genetic marker to identify vaccinated animals through PCR. To do so, faecal samples from each group were pooled and collected periodically and bacterial genetic material was extracted. Oligonucleotide DIVA-1 was designed so that the 5´end of the oligonucleotide hybridizes to a DNA sequence downstream *sen4316* whilst the 3´end of the oligonucleotide hybridizes to a DNA sequence upstream *sen4316* (Fig 6B). Oligonucleotide DIVA-2 hybridizes to a DNA sequence downstream *sen4317* (Fig 6B). PCR conditions were designed in such a way that amplification only took place if the ΔXIII genome acted as template DNA. On the other hand, amplification of the *Salmonella* specific gene *invA* with oligonucleotides invA-fw and invA-rv was used as an internal positive control in all samples. Faecal samples proceeding from animals infected with the wild type strain resulted *invA* positive, DIVA negative, while samples proceeding from vaccinated animals resulted *invA* positive, DIVA positive (Fig 6B).

These results confirmed that ΔXIII can be considered a DIVA vaccine since the GGDEF protein SEN4316 enables discrimination of vaccinated animals either by immunological or by molecular non sophisticated approaches.

### Survival of ΔXIII in the environment

One important drawback of live attenuated vaccines is that the strain, once excreted by vaccinated animals, may persist for long periods in the environment. To determine whether this might be a handicap to the use of ΔXIII strain, phenotypic traits involved in environmental persistence, such as resistance to desiccation in the absence of nutrients and biofilm formation were assessed.

**Fig 7:**
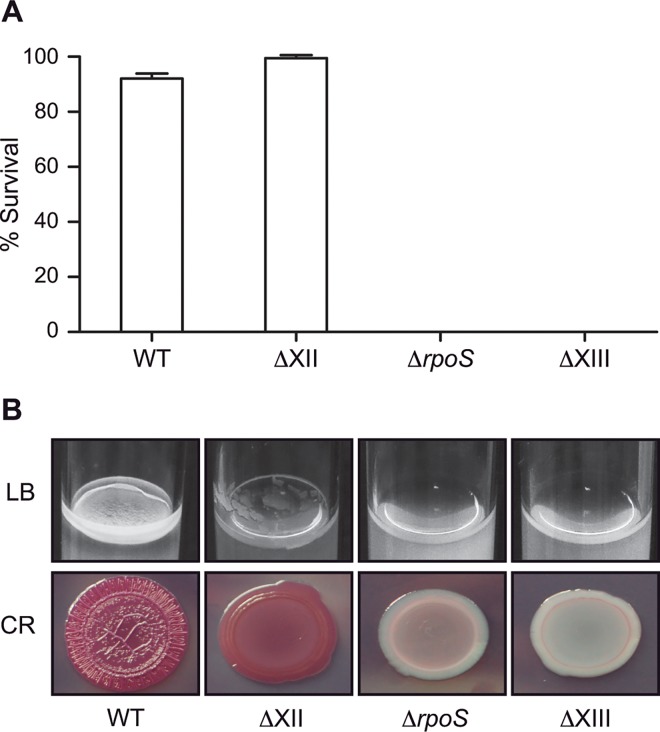
ΔXIII strain is unable to form a biofilm and does not survive after twelve days of desiccation in the absence of nutrients. (A) Survival of the wild type strain, new ΔXII (absence of c-di-GMP), the single Δ*rpoS* mutant and the vaccine candidate ΔXIII after twelve days of desiccation. Surviving bacteria were enumerated by viable plate counts, and their numbers were compared with those of initial inocula, which defined 100% survival. Means and standard deviations of results from three independent experiments are shown. (B) Biofilm formation capacity of the same strains. Biofilm phenotypes were visualized after growth in LB medium conditions and on congo red agar plates.

Results showed that almost 100% of wild type bacteria remained viable after 12 days under waterless conditions. On the contrary, both Δ*rpoS* and ΔXIII strains showed to be dramatically sensitive to desiccation, since, at the end of the experiment, there were not any live bacteria left (Fig 7A). Biofilm formation capacity was analyzed by incubating bacteria in LB broth under static and room temperature conditions for 72 hours and also, by analyzing colony morphology on plates containing the Congo Red dye (Fig 7B). Δ*rpoS* and ΔXIII strains did not form a biofilm in LB and showed a “saw” (smooth and white) morphotype, which corresponds with the inability to produce cellulose and fimbriae, the main components of the *Salmonella* biofilm matrix, and thus a total absence of multicellular behavior.

These results demonstrate that ΔXIII might be easily eliminated from the environment when excretion by vaccinated animals occurs.

## Discussion

C-di-GMP is recognized as a universal bacterial second messenger implicated in the regulation of a large number of cellular functions, including cell cycle progress, differentiation, biofilm formation and dispersion, motility and virulence. As regards virulence, it is generally assumed that high levels of c-di-GMP in bacterial cells promote biofilm formation and the establishment of chronic infections, whilst low c-di-GMP levels promote virulence factor synthesis and thus, acute infections [17,33,34]. In contrast to this general view, our results with new ΔXII strain, which lacks all GGDEF domain proteins and thus cannot make c-di-GMP, have demonstrated that, in *Salmonella*, absence of c-di-GMP signaling is detrimental for the development of an acute infection. Virulence decrease shown by ΔXII strain was subtle but consistent enough to indicate that either an specific GGDEF domain protein or a certain c-di-GMP related phenotype or c-di-GMP itself might be required at some stage of the *Salmonella* acute infection. These results are in line with recent evidences that are revealing a more complicated picture about the relationship of the c-di-GMP network and virulence than expected. Individual mutants in not only c-di-GMP degrading enzymes (phosphodiesterases; PDEs) but also synthesizing enzymes (diguanylate cyclases; DGCs) have shown to display attenuation in infection models [35–37], suggesting that opposite c-di-GMP-metabolizing processes might play a role at different disease stages [17]. Also, putative DGCs and PDEs with no c-di-GMP metabolizing activity have been described to affect virulence [38–42]. Accordingly, virulence defect of ΔXII strain might be explained by the fact that several *Salmonella* GGDEF domain proteins harbor a degenerated consensus signature and might somehow control virulence independently of c-di-GMP levels. With respect to the participation in virulence of a particular c-di-GMP controlled phenotype, we showed in a previous study that production of cellulose is not involved in the virulence of *S*. Enteritidis in BALB/c mice infected orally or intraperitoneally [25]. However, a very recent report by Pontes et al. [43] has demonstrated that *Salmonella* makes cellulose inside macrophages and that its synthesis hinders *Salmonella* replication inside host cells. Thus, a cellulose mutant strain was hypervirulent in C3H/HeN mice injected intraperitoneally. In any case, results of both studies do not correspond with results obtained here which show that ΔXII strain, that cannot synthesize cellulose, is less virulent than the wild type strain. Hence, virulence defect of ΔXII strain cannot be attributed to a lack of cellulose synthesis. With regard to the direct contribution of c-di-GMP to virulence, it is known that it binds to the eukaryotic proteins STING and DDX41 [18–21], leading to induction of type I interferons and thus, apparently contributing to successful pathogen elimination. This fact, again, does not agree with ΔXII strain being less virulent than the wild type strain. On the contrary, ΔXII virulence attenuation might be related to a very recent research of Li et al. [44] finding that c-di-GMP directly binds to human siderocalin (LCN2). LCN2 sequesters bacterial siderophores to prevent iron acquisition and inhibit bacterial growth under iron-limited conditions [45,46]. Li et al. showed that c-di-GMP competes with bacterial siderophores to bind to LCN2, therefore, preventing LCN2 from inhibiting bacterial growth. These results indicated that c-di-GMP interferes with LCN2 to ensure bacterial survival during infection and that inhibition of c-di-GMP production should attenuate virulence. Further detailed analysis of the contribution of the c-di-GMP signaling network to *Salmonella* virulence will be required to figure out the reasons why ΔXII strain presents an attenuated phenotype.

Once we cleared up the effect of the absence of c-di-GMP signaling in *Salmonella* virulence, and with the final aim of constructing a novel vaccine candidate, we performed a mutation of the *rpoS* gene in ΔXII, giving rise to strain ΔXIII. Absence of all GGDEF domain proteins and also of RpoS had a synergistic attenuating effect that made ΔXIII extremely safe and capable to protect vaccinated mice from a lethal dose challenge of a wild type *S*. Typhimurium strain. The cross-talk between T and B cells is of fundamental importance for the establishment of solid acquired immunity to salmonellosis [8]. Accordingly, mice protection with ΔXIII strain was found to occur via different immune responses including *Salmonella*-specific IgGs that showed a moderate opsonic activity and high levels of the pro-inflammatory cytokines IFN-γ, TNF-α, IL-2, IL-17 and also of the anti-inflammatory cytokine IL-10. IFN-γ has a crucial role for clearing infections caused by intracellular bacteria, given that its major effect is macrophage recruiting and that activation and high titters of this cytokine generally implies a strong immune response that is accompanied by T-lymphocyte expansion [47,48]. Thus, mice treated with anti-IFN-γ antibodies and also, mice and humans genetically deficient in immunity mediated by IFN-γ are highly susceptible to *Salmonella* [49–51]. With respect to IL-17, it is emerging as a central element of mucosal immunity to microbial challenge since it enhances basic innate barrier defenses such as antimicrobial peptide production and neutrophil recruitment [52]. Accordingly, *S*. Typhimurium-infected IL-17-receptor-A-deficient mice have lower amounts of inflammatory cytokines, less neutrophil recruitment and increased bacterial translocation to the spleen and mesenteric lymph nodes [53]. On the other hand, IL-10 is a well-characterized anti-inflammatory cytokine that inhibits synthesis of Th1 cytokines and proliferation of T cells, preventing the detrimental consequences of systemic inflammation [54]. The presence of IL-10 in ΔXIII immunized animals suggests that the immune response was tightly balanced [55,56] and that ΔXIII might exploit the immunosupresive effects of IL-10 to persist in mice organs, eliciting a long-lasting immunity [57].

Up to date there is no ideal vaccine available for control of salmonellosis in farm animals. Such a vaccine should be cheap, minimally reactive and preferably live and invasive but still safe to induce strong immunity [58]. Furthermore, the use of attenuated vaccines as veterinary drugs can only be recommended if differentiation of vaccinated from infected animals (DIVA) is feasible in a basic bacteriology laboratory [58,59]. In response to this need, antigenic, genetic and phenotypic markers have been incorporated into or eliminated from attenuated vaccines [60,61]. However, bacterial “tagging” is normally achieved through genetic engineering methods and serious concerns are being raised about the effects that genetically modified microbes (GMMs) may have if massively used or released into the environment [62]. In order to avoid the use of antibiotic markers, ΔXIII strain is the result of thirteen consecutive deletion steps in which an allelic exchange strategy with *sacB* as the counterselectable marker was used [16,22]. Thus, ΔXIII does not contain any trace of exogenous DNA and is indistinguishable from a naturally occurring mutant, fact that eludes its classification as a GM organism according to the European Directive [EC] 2001/18. Amongst the thirteen candidates to become a negative selectable marker, we found several reasons for choosing the GGDEF protein SEN4316. In contrast to other GGDEF proteins, SEN4316 is conserved in all *S*. *enterica* serovars and thus, it can be considered a “broad spectrum” marker since seroconversion is likely to occur upon infection of animals with any *Salmonella* serovar, thereby activating the DIVA function [59]. On the other hand, *E*. *coli* and other gram negative species lack this member of the GGDEF family (data not shown). We designed a SEN4316 based ELISA that allowed the serological discrimination of vaccinated from infected mice. In addition, and since such DIVA strategy identifies infected animals, we developed a very simple molecular method to positively identify vaccinated animals. However, its application would be restricted to the period of time in which the vaccine is present in faeces.

Because live vaccines can be potentially excreted into the environment by immunized animals, another condition of increasing interest in vaccine development is the easiness to eliminate it from the environment [3,63]. Undoubtedly, this is one of the strengths when considering ΔXIII strain as a live vaccine candidate since it is totally incapable of synthesizing a protective biofilm matrix and lacks RpoS, a regulator that is indispensable to mount an adequate response against environmental stresses. Accordingly, less than two weeks were sufficient to end up with 100% of ΔXIII bacteria under waterless conditions.

*Salmonella* mutants of reduced virulence that still display high immunogenicity have already been described [3–5,10]. We propose that the c-di-GMP signaling and RpoS defective *Salmonella* strain described here, which shows a delicate safety-immunogenicity balance, promising DIVA features and can be easily eliminated from the environment is a noticeable new candidate to include in this list. Future work will be conducted to analyze its potential use in field vaccination trials.

## Supporting Information

S1 Fig Representative IFN-γ and TNF-α/CD4 staining for ΔXIII stimulated splenocytes. 28 days after immunization, splenocytes from control and ΔXIII immunized mice were plated and stimulated for 6 hours with medium, 10^6^ or 10^7^ cfu of heat-killed ΔXIII bacteria. Cells were surface stained with anti-CD4-FITC antibody and intracellularly stained with anti IFN-γ-PE and anti-TNF-α-PE-Cy7 mAbs. Shown are percentages of IFN-γ and TNF-α cells out of total CD4+ T cells.
(EPS)

S1 Table Strains used in this study. (DOCX)
