# Supplementary material for: Evaluation of a Salmonella Strain Lacking the Secondary Messenger C-di-GMP and RpoS as a Live Oral Vaccine
Source: PLoS One. 2016 Aug 18;11(8):e0161216. doi: 10.1371/journal.pone.0161216 (PMC4990191; doi:10.1371/journal.pone.0161216)
Supplement: S1 Table — (DOCX) [file pone.0161216.s002.docx]

S1 Table. Strains used in this study

| **Strain** | **Genotype** | **Degree of attenuation** | **MIC^a^** | **Reference or source** |
| --- | --- | --- | --- | --- |
| ***S*. Enteritidis** |  |  |  |  |
| 3934 | Wild-type clinical isolate | **-** | 54 | [24]  [25] |
| ΔXII | 3934 Δ*adrA* Δ*sen1023* Δ*yeaJ* Δ*yciR* Δ*yegE* Δ*yfiN* Δ*yhda* Δ*sen3222* Δ*yhjK* Δ*sen2484* Δ*yfeA* Δ*sen4316* | **+** | 1324 | [22] |
| ΔXII Km^R^ Tc^R^ | 3934 Δ*adrA* Δ*sen1023 yeaJ::*Tc^R^ Δ*yciR* Δ*yegE* Δ*yfiN* Δ*yhda* Δ*sen3222* Δ*yhjK* Δ*sen2484 yfeA::* Km^R^ Δ*sen4316* | **+** | 3664 | [22] |
| ΔXIII | 3934 Δ*adrA* Δ*sen1023* Δ*yeaJ* Δ*yciR* Δ*yegE* Δ*yfiN* Δ*yhda* Δ*sen3222* Δ*yhjK* Δ*sen2484* Δ*yfeA* Δ*sen4316* Δ*rpoS* | **+++** | 1330 | This study |
| Δ*rpoS* | 3934 *rpoS*::Apr^R^ | **++** | 2101 | This study |
| ***S*. Typhimurium** |  |  |  |  |
| *S.* Typhimurium 143/09 | Wild-type clinical isolate | **-** | 1201 | Centro Nacional de Microbiología |
| ***E.coli*** |  |  |  |  |
| XL1-Blue | *recA1* *endA1* *gyrA96* *thi-1 hsdr*17 *supE*44 *rel*A1 *lac* [F´*proAB lacl*^q^ Z Δ*M15* Tn10 (Tc^R^)] |  | 797 | Stratagene |
| MC4100 Apr^R^ | MC4100 F´tet ΔtraD::aac |  | 1087 | [27] |
| *E.coli* BL21 C43(DE3) | *huA2 [lon] ompT gal (λ DE3) [dcm] ∆hsdS λ DE3 = λ sBamHIo ∆EcoRI-B int::(lacI::PlacUV5::T7 gene1) i21 ∆nin5* |  | 1637 | [31] |

a. Number of each strain in the culture collection of the Laboratory of Microbial Biofilms, Instituto de Agrobiotecnología (Idab)
